# Supplementary material for: Physiological Responses to Combat Sports in Metabolic Diseases: A Systematic Review
Source: J Clin Med. 2022 Feb 18;11(4):1070. doi: 10.3390/jcm11041070 (PMC8878771; doi:10.3390/jcm11041070)
Supplement: Supplementary file 1 [file jcm-11-01070-s001.zip › jcm-1593830-supplementary.pdf]

Supplementary Material

Table S1. Search terms used for literature research.

Combination of the topics *"combat sport"* and *"metabolic disease"*	combined search-term of all topics was used, split by the *"relevant marker"* key words
1. ((diabetes mellitus, type 1) AND (martial arts) NOT (Tai Chi))<br>2. ((diabetes mellitus, type 1) AND (combat sports) NOT (Tai Chi))<br>3. ((diabetes mellitus, type 1) AND (full contact) NOT (Tai Chi))<br>4. ((diabetes mellitus, type 1) AND (full contact [tw]) NOT (Tai Chi))<br>5. ((diabetes mellitus, type 1) AND (light contact) NOT (Tai Chi))<br>6. ((diabetes mellitus, type 1) AND (light contact [tw]) NOT (Tai Chi))<br>7. ((diabetes mellitus, type 2) AND (martial arts) NOT (Tai Chi))<br>8. ((diabetes mellitus, type 2) AND (combat sports) NOT (Tai Chi))<br>9. ((diabetes mellitus, type 2) AND (full contact) NOT (Tai Chi))<br>10. ((diabetes mellitus, type 2) AND (full contact [tw]) NOT (Tai Chi))<br>11. ((diabetes mellitus, type 2) AND (light contact) NOT (Tai Chi))<br>12. ((diabetes mellitus, type 2) AND (light contact[tw]) NOT (Tai Chi))<br>13. ((metabolic syndrome) AND (martial arts) NOT (Tai Chi))<br>14. ((metabolic syndrome) AND (combat sports) NOT (Tai Chi))<br>15. ((metabolic syndrome) AND (combat sports[tw]) NOT (Tai Chi))<br>16. ((metabolic syndrome) AND (full contact) NOT (Tai Chi))<br>17. ((metabolic syndrome) AND (full contact [tw]) NOT (Tai Chi))<br>18. ((metabolic syndrome) AND (light contact) NOT (Tai Chi))<br>19. ((metabolic syndrome) AND (light contact[tw]) NOT (Tai Chi))<br>20. ((obesity) AND (martial arts) NOT (Tai Chi))	1. (diabetes mellitus, type 1) OR (diabetes mellitus, type 2) OR (metabolic syndrome) OR (obesity) OR (diabesity) AND (martial arts[tw]) OR (combat sports[tw]) OR (full contact[tw]) OR (light contact[tw]) AND (glucose)<br>2. (diabetes mellitus, type 1) OR (diabetes mellitus, type 2) OR (metabolic syndrome) OR (obesity) OR (diabesity) AND (martial arts[tw]) OR (combat sports[tw]) OR (full contact[tw]) OR (light contact[tw]) AND (MET)<br>3. (diabetes mellitus, type 1) OR (diabetes mellitus, type 2) OR (metabolic syndrome) OR (obesity) OR (diabesity) AND (martial arts[tw]) OR (combat sports[tw]) OR (full contact[tw]) OR (light contact[tw]) AND (lactate)<br>4. (diabetes mellitus, type 1) OR (diabetes mellitus, type 2) OR (metabolic syndrome) OR (obesity) OR (diabesity) AND (martial arts[tw]) OR (combat sports[tw]) OR (full contact[tw]) OR (light contact[tw]) AND (VO2)<br>5. (diabetes mellitus, type 1) OR (diabetes mellitus, type 2) OR (metabolic syndrome) OR (obesity) OR (diabesity) AND (martial arts[tw]) OR (combat sports[tw]) OR (full contact[tw]) OR (light contact[tw]) AND (heart rate)

21. ((obesity) AND (combat sports) NOT (Tai Chi))<br>22. ((obesity) AND (combat sports[tw]) NOT (Tai Chi))<br>23. ((obesity) AND (full contact) NOT (Tai Chi))<br>24. ((obesity) AND (full contact[tw]) NOT (Tai Chi))<br>25. ((obesity) AND (light contact) NOT (Tai Chi))<br>26. ((obesity) AND (light contact[tw]) NOT (Tai Chi))<br>27. ((diabesity) AND (martial arts) NOT (Tai Chi))<br>28. ((diabesity) AND (combat sports) NOT (Tai Chi))<br>29. ((diabesity) AND (full contact) NOT (Tai Chi))<br>30. ((diabesity) AND (light contact) NOT (Tai Chi))<br>31. ((diabetes mellitus, type 1) OR (diabetes mellitus, type 2) OR (metabolic syndrome) OR (obesity) OR (diabesity)) AND ((martial arts[tw]) OR (combat sports[tw]) OR (full contact[tw]) OR (light contact[tw])NOT (Tai Chi))	
